# CING: an integrated residue-based structure validation program suite

**DOI:** 10.1007/s10858-012-9669-7

**Published:** 2012-09-18

**Authors:** Jurgen F. Doreleijers, Alan W. Sousa da Silva, Elmar Krieger, Sander B. Nabuurs, Christian A. E. M. Spronk, Tim J. Stevens, Wim F. Vranken, Gert Vriend, Geerten W. Vuister

**Affiliations:** 1 CMBI, Radboud University Medical Centre, Geert Grooteplein 26-28, 6525 GA Nijmegen, The Netherlands; 2 UniProt, European Bioinformatics Institute, Hinxton, Cambridge, CB10 1SD UK; 3 YASARA Biosciences GmbH, Wagramer Strasse 25/3/45, 1220 Vienna, Austria; 4 Spronk NMR Consultancy UAB, Palangos gatvė 4, 01402 Vilnius, Lithuania; 5 Department of Biochemistry, University of Cambridge, 80 Tennis Court Road, Cambridge, CB2 1GA UK; 6 Department of Structural Biology, VIB, Building E, 4th Floor, Pleinlaan 2, 1050 Brussels, Belgium; 7 Structural Biology Brussels, Vrije Universiteit Brussel, Building E, 4th Floor, Pleinlaan 2, 1050 Brussels, Belgium; 8 Department of Biochemistry, University of Leicester, Henry Wellcome Building, Lancaster Road, Leicester, LE1 9HN UK

**Keywords:** NMR, Structure validation, PDB, Errors, Quality, Protein structure

## Abstract

We present a suite of programs, named CING for Common Interface for NMR Structure Generation that provides for a residue-based, integrated validation of the structural NMR ensemble in conjunction with the experimental restraints and other input data. External validation programs and new internal validation routines compare the NMR-derived models with empirical data, measured chemical shifts, distance- and dihedral restraints and the results are visualized in a dynamic Web 2.0 report. A red–orange–green score is used for residues and restraints to direct the user to those critiques that warrant further investigation. Overall green scores below ~20 % accompanied by red scores over ~50 % are strongly indicative of poorly modelled structures. The publically accessible, secure iCing webserver (https://nmr.le.ac.uk) allows individual users to upload the NMR data and run a CING validation analysis.

**Electronic supplementary material:**

The online version of this article (doi:10.1007/s10858-012-9669-7) contains supplementary material, which is available to authorized users.

## Introduction

Nuclear Magnetic Resonance (NMR) Spectroscopy is the second most important tool for the structure determination of biomolecules at the atomic level. Approximately 12 % of all ~82,000 deposited biomolecular structures in the Worldwide Protein Databank, wwPDB (Berman et al. 2003) have been solved by NMR. This percentage increases to ~25 % if only unique folds are considered (Laskowski 2003). The steady increase in the number of biomolecular structures solved by NMR in the wwPDB originates both from the increased world-wide capacity, e.g. as result of the efforts of the structural genomics consortia, as well as from improved technology in several, often automated stages of the structure determination process itself (Güntert 2009; Rieping et al. 2007). For NMR, it has been reported that the data acquisition and data analysis, and the subsequent structure determination of a moderately-sized protein by NMR could take as little as 1–9 days (Liu et al. 2005).

The detection of a series of fraudulent X-ray structures at the end of 2009 brought the topic of validation back to the forefront (Baker et al. 2010). The PDB NMR data has only recently been fully remediated and validated (Doreleijers et al. 2009; Henrick et al. 2008) including the corrections to the hydrogen atom nomenclature and geometry (Doreleijers et al. 1999). CING presents the tools for authors, referees, and end-users to validate NMR structures in a comprehensive and integrated way.

For NMR, as with any other experimental technique, it is imperative that the transformation of experimental data into resulting structures occurs according to well-described, reproducible procedures. For high-resolution NMR, this transformation typically involves three steps: raw NMR data are first processed by Fourier transformation and peak picked. Next, the resulting spectral data, such as resonance frequencies, peaks, and fine-structure are converted into structural restraints. In a third step, a computational algorithm transforms these restraints into an ensemble of conformers. This latter step often involves a simulated annealing molecular dynamics calculation. NMR structure calculation programs such as AMBER (Case et al. 2005), CYANA (Güntert 2004), ARIA (Habeck et al. 2004) and Xplor-NIH (Schwieters et al. 2006) typically provide information regarding the agreement between the NMR ensemble and the experimental restraints, as well as some rudimentary information regarding the quality of the structure ensemble. A more detailed quality analysis is typically performed using external programs such as PROCHECK_NMR/AQUA (Laskowski et al. 1996), Molprobity (Lovell et al. 2003), WHAT_CHECK (Hooft et al. 1996b), and sometimes Model Quality Assessment Programs (MQAPs) (McGuffin 2007). More recently, a visual validation web server, called NMR Constraints Analyser, has been presented (Heller and Giorgetti 2010) that focuses on the validation of the distance restraints. Other programs such as PSVS (Bhattacharya et al. 2007), GLM (Bagaria et al. 2012) and ResProx (http://www.resprox.ca) have combined several of the common tools with their own specific checks.

Surveys by ourselves (Doreleijers et al. 1998; Hooft et al. 1996b; Nabuurs et al. 2005, 2006), and others e.g. (Bhattacharya et al. 2007; Snyder et al. 2005) indicated that the commonly accepted protocols in NMR for validation of the structure ensemble do not always detect misfolded structures or other serious problems. Our analyses even showed that a wrongly folded structure (PDB code 1tgq, now redeposited as 2b95) can be refined to such an extent that commonly reported NMR parameters for structural quality, such as restraint violations and Ramachandran plot scores, will not flag it as having serious problems (Nabuurs et al. 2006). Likewise, structure refinement against a single set of experimental residual dipolar couplings (RDCs) can also yield seemingly good structures without violations, even if the experimental data were completely randomized (Bax and Grishaev 2005). These examples illustrate the need for a more sophisticated NMR structure quality validation approach.

Our analysis of the obsolete PDB entry 1tgq indicated that an underlying cause of the problems was the neglect of the specific nature of the NMR data, which for the most part are highly local. As a result, the NMR ensemble can contain both well-resolved and problematic areas. Hence, the structural quality cannot be captured in a single parameter that describes an overall structure property, because such a value will be the average over the good and the bad parts. Instead, we advocate a residue-oriented approach to properly account for this variability. Here, we present a suite of programs based upon this philosophy, named CING (pronounced as ‘king’) for Common Interface for NMR Structure Generation that provides for a residue-based, integrated validation of the structure ensemble together with the experimental restraints and other data. The program is optimized for, but not limited to, NMR-derived biomolecular structures.

The CING validation will implement and follow the forthcoming wwPDB NMR validation task force recommendations. The iCing Virtual Machine web server provides for easy, anonymous access to the CING validation suite for both human users and automated submission by external programs. The server is available from the WeNMR Virtual Research Community’s web portal to the grid at http://wenmr.eu (Wassenaar et al. 2011) and via https://nmr.cmbi.ru.nl/icing/ or https://nmr.le.ac.uk.

## Methods

### CING program philosophy and design

The information derived from existing structure validation programs was studied to see how and to what extent this information was typically used and reported on by the NMR community (Markley et al. 1998). This analysis showed that existing programs suffered from a number of fundamental and practical drawbacks that strongly limited their usage. The practical drawbacks included (a) programs being out-dated and no longer actively developed, (b) not being suitable for NMR often because of their inability to adequately handle the multiple models contained in the NMR ensemble and (c) programs being difficult to run and install. At the more fundamental level, the knowledge obtained from the existing programs was typically hard to analyse as well as difficult to automatically integrate with the rest of the data; hence a combination of validation knowledge from different sources was generally not used. In addition, the direct relation to the experimental data often would be absent and essential functionalities for proper structure evaluation were missing.

#### Data structures

**Fig. 1:**
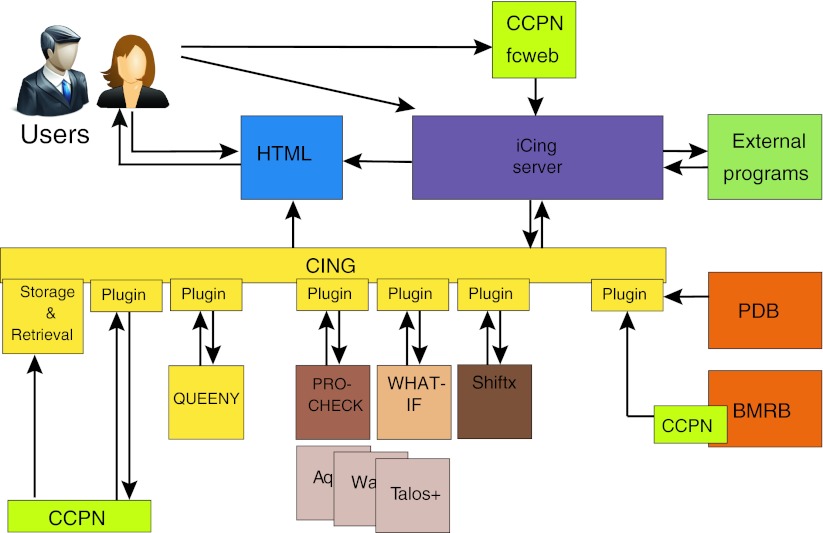
Schematic outline showing the data flow and software components involved in CING (*yellow boxes*). External programs interfaced to CING, CCPN services/APIs and wwPDB repositories are represented in *brown*-*shades*, *green* and *dark*-*orange*, respectively. External programs (*olive green*) can access the iCing web user interface (*purple*) through the dedicated iCing robot

In order to be able to successfully handle very diverse types of information, we implemented in CING a data structure that represents all NMR elements, such as resonances, peaks, restraints, molecules with their chains, residues, atoms and coordinates, as well as the results obtained from validation routines and we defined all the connections between all these elements. The core layer of CING (Fig. 1) implements this data structure and the data storage including the reference data and the API to access it. The total set of all data, i.e. experimental data such as chemical shift and peak, distance and dihedral restraint, coordinate and validation data, are stored as an integrated set, called a project. An XML dialect, denoted as Simple Markup File (SML) format, is used for internal storage of the reference information as well as the CING project data. Isolated pieces of code, called plugins, interface to the external programs that CING uses for its analyses (cf. Table 1). Each program’s plugin exports the data to the desired format of the external program, executes the program, and imports the results back into the CING data framework. In all cases, the input and all output data are retained within the CING project.

**Table 1:** External programs interfaced by CING

Program	O	Version	References	Url (http)
CING	−	r1136	N.a.	nmr.cmbi.ru.nl/cing
Cython	−	0.15	N.a.	cython.org
Ghostscript	−	9.04	N.a.	ghostscript.sf.net
ImageMagick	−	6.7.3-1	N.a.	imagemagick.org
MatplotLib	−	1.0.1	Hunter (2007)	matplotlib.sf.net
Python	−	2.7	Lutz (2001)	python.org
Analysis	+	2.1.5	Vranken et al. (2005)	www.ccpn.ac.uk/ccpn/software/ccpnmr-analysis
AQUA	+	3.2 (r15)	Laskowski et al. (1996)	nmr.cmbi.ru.nl/~jd/aqua
CCPN	+	r6249	Vranken et al. (2005)	www.ccpn.ac.uk
DSSP	+	2010-04-01	Hooft et al. (1996a)	swift.cmbi.ru.nl/gv/dssp
MolMol	+	2K.2	Koradi et al. (1996)	No longer supported
PROCHECK-NMR	+	3.5.4	Laskowski et al. (1996)	www.ebi.ac.uk/thornton-srv/software/PROCHECK/
PyMol	+	1.2r1	DeLano and Bromberg (2004)	pymol.org
Queeny	+	r1076	Nabuurs et al. (2003) (original)	www.cmbi.kun.nl/software/queen (original)
SHIFTX	+	1.1.0	Zhang et al. (2003)	shiftx.wishartlab.com
Talos+	+	1.01	Shen et al. (2009)	spin.niddk.nih.gov/NMRPipe/talos
VASCO	+	r6249	Rieping and Vranken (2010)	www.ebi.ac.uk/pdbe/nmr/vasco
Wattos	+	r154	Doreleijers et al. (2005)	nmr.cmbi.ru.nl/~jd/wattos
WHAT_CHECK	+	2010-08-16	Hooft et al. (1996b)	swift.cmbi.ru.nl/gv/whatcheck
Xplor-NIH	+	2.26	Schwieters et al. (2006)	nmr.cit.nih.gov/xplor-nih
YASARA	+	11.6.1	Joosten et al. (2011)	www.yasara.org

The maximum version of any instalment is listed. For example, the Python version for CING is 2.7 although version 2.5 is also supported. The optional status (second column labelled O) is based on the most basic CING functionality excluding plugins but including plotting features

### Implementation

The CING software development uses the Google Code repository at http://code.google.com/p/cing. Elements of *Extreme programming* (Beck and Andres 2004) such as code review, and daily commits (1,185 revisions to date) are an essential part of the design and the daily maintenance. CING is mostly implemented in Python, an open-source, high-level, object-oriented, interpreted language (Lutz 2001; Millman and Aivazis 2011). A small fraction of the code is implemented in C using the cython Python-to-C interface because of speed reasons (Behnel et al. 2011). Two-dimensional graphics were implemented using the matplotlib library (http://matplotlib.sourceforge.net). Table 1 lists the software tools and external programs that have thus far been incorporated in CING code. CING is freely available at Google under a GNU Lesser General Public License. Virtual Machine images are available upon request from the authors.

#### Data conversion

The ability to accommodate a wide array of data types and formats is unfortunately still crucial for any NMR structure validation program. Relevant formats, such as those of CCPN and CYANA, can be handled by CING internally through the use of program-specific plugin converters (see Fig. 1 and described above). The CCPN data format captures an enormous variety of data and has been well-used and tested in many laboratories around the world. The on-line tool based on the CCPN FormatConverter for conversion to and from the CCPN format is actively maintained (Vranken et al. 2005) (available at: http://webapps.ccpn.ac.uk/fcweb). Hence, data in the CCPN format has our preference for interaction with CING.

#### Reference data organization

The CING program is inherently ‘NMR-aware’. The data related to the molecular topology of residues, reference chemical shifts of atoms, the notion of pseudoatoms, etc. resides in so-called database per-residue SML files. This reference database includes all common amino and nucleic acids, protonation variants, and several special entities such as water and ions. The reference data includes definitions for dihedral angles and atomic properties such as type, spin, and BioMagResBank (BMRB) derived average and standard deviations of chemical shift values (Markley et al. 2008). A new entry into this database is created on the fly when the CING importing routines encounter a non-canonical residue. This entry is then stored within the CING project. The nomenclature of CING atoms and residues follows IUPAC standards (Markley et al. 1998) with extensive conversion facilities for other nomenclatures, called ‘conventions’, such as CCPN, CYANA, X-PLOR, and their dialects. Alternative atom and residue names can be used as aliases.

#### Report pages

CING combines the output of its own routines and the external programs and generates a comprehensive report in the form of interactive HTML pages. The hierarchical organization of the pages reflects the aggregation levels (project, molecule, residues, peaks, restraints etc.). The pages are extensively cross-linked for easy navigation. The user can interact with the report in several ways using Web 2.0 Javascript functionality such as provided by JQuery (http://jquery.com) and a JQuery plugin called Datatables (http://www.datatables.net). All entities within the CING project are evaluated and issued a three-way ROG-assessment: problems (red), potential problems (orange), or no problems found (green) (cf. Table 2). The ROG colouring is used throughout the HTML pages when referring to the different entities.

**Table 2:** ROG scoring criteria

Entity	Program	Property	Orange	Red
Molecule	CING	ROG^a^	%G ≤ 20 + %R	%G ≤ %R − 20
Chain	CING	Propagated from residue entities^b^		
Residue	CING	Omega deviation (°)^c^	9.4	14.1
Residue	CING	D1D2 plot (Z)^d^	−1.0	−0.8
Residue	WHAT_CHECK	Ramachandran (Z)^d^	−1.0	−1.3
Residue	WHAT_CHECK	Janin plot (Z)^d^	−0.9	−1.2
Residue	PROCHECK_NMR	G-factor^e^	−1.0	−1.3
Atom	CING	Propagated from CS entities^b^		
Peak-list	CING	Propagated from peak entities^b^		
Peak	CING	Linking^f^	Unassigned atom	
Peak	CING	CS assignment (Z)^g^	1	2
CS-list	CING	Propagated from CS entities^b^		
CS	CING	CS assignment (Z)^h^	3	
CS	CING	No coordinates	No coordinates	
CS	CING	Leucine side chain^i^		Inconsistency
CS	CING	Proline omega^j^		Inconsistency
CS	CING	Assignment issues^f^	Various	
DR-list	CING	Propagated from DR entities^b^		
DR	CING	Max. violation (Å)^k,l^	0.3	0.5
DR	CING	RMS violation (Å)^l^	0.15	0.3
DR	CING	Presence atoms		No coordinates
DH-list	CING	Propagated from DH entities^b^		
DH	TALOS+/CING	Max. violation (°)^l^	3	10
DH	CING	RMS violation (°)^l^	3	5
DH	CING	Presence atoms		No coordinates
RDC-list	CING	–^m^		
RDC	CING	–^m^		

ROG scoring criteria at each entity level. The Program column denotes the program used for the assessment. See footnotes for the rationales of the cut off criteria values used. CS denotes chemical shift, DR distance restraint, DH dihedral angle restraint and RDC residual dipolar coupling restraint

^a^The residue critiques (line items 3 through 7) propagate to the molecule level by evaluating the listed inequalities for orange and red scoring, using percentages of residues with a red (%R) and green (%G) ROG score. Only the well-defined residues, as determined by the CV-criterion (see methods) were included in the Molecule criterion. Results from data entities are not included in the Molecule ROG score

^b^Entity obtains the worst propagated ROG score. The residue critiques are the only items that cascade up to the Molecule level, in other words, e.g. the experimental data critiques do currently not affect the overall ROG score

^c^The omega deviation is calculated as an average over the ensemble with the references values for *cis* and *trans* peptide bonds values taken from (Wilson et al. 1998). The cut offs are 3 and 4 SD removed from those averages

^d^The unit for these criteria is the number of standard deviations denoted Z. The cut off were determined by manually examining a large number of examples. See text for a short introduction to the D1 and D2 dihedrals

^e^Manually determined cut off

^f^Various assignment issues are scored orange for e.g. the presence of multiple assignments, missing assignments and invalid stereospecific assignments

^g^The standard deviation for the chemical shift assignment of peaks was assumed to be a uniform 0.01 ppm for ^1^H and 0.15 ppm for all other nucleii ^15^N, ^13^C, and ^31^P. The uncertainties on the observed CS have not been considered

^h^The CS are flagged with respect to the BMRB derived database values

^i^The Leucine CS are compared for consistency with the side chain conformation (Doreleijers et al. 2011; Mulder 2009)

^j^The Proline CS are compared for consistency with the peptide bond conformation as described in the text as based on (Shen and Bax 2009)

^k^The maximum restraint violation in any member of the ensemble

^l^Commonly used cut off, e.g. in Xplor-NIH analysis scripts

^m^RDCs are currently not validated and do not receive a ROG score

#### Imagery

CING is integrated with the molecular graphics programs: YASARA (Joosten et al. 2011), PyMol (DeLano and Bromberg 2004), MOLMOL (Koradi et al. 1996) and JMol (Herráez 2006). CING can instruct each of these programs *via* macros to render properties such as the per-residue ROG score colouring onto the backbone.

#### Web services

iCing is a secure web portal (https://nmr.cmbi.ru.nl/icing/) to the CING server that allows users to validate their own data. The iCing web portal currently touts three input formats that can be used to submit complete projects (coordinates, experimental data, and restraints): CING, CCPN, and CYANA as well as the PDB format for importing a structure ensemble without additional data. The API to the CING-formatted data is described in the Google-code repository. iCing also serves as a interface to CING for third-party applications. An iCing robot allows for automated upload of project data and the return of XML formatted validation results. The iCing front-end is implemented using the Google Web Toolkit technology.

### CING tools for experimental data analysis

#### Chemical shifts

Various potential assignment issues are evaluated, such as the presence of multiple assignments, missing assignments, and inconsistent pseudo-atom and/or stereo-specific assignments. In addition, the chemical shifts are compared to the BMRB-derived distributions and compared to the back-calculated values using the SHIFTX program (Zhang et al. 2003).

#### Peaks

Peaks typically represent an abstracted stage of the experimental data and typically are neither deposited with the structure ensemble nor retained otherwise. CING stores and analyses peak entities for consistency with valid assignments.

#### Restraint analyses

The distance and dihedral angle restraints are validated to show the (RMS) violations in the ensemble and counts of models in which a violation occurs above the thresholds commonly used (lower-bound violations and 0.1, 0.3, and 0.5 Å for upper-bound violations of the distance restraints and 1, 3, and 5° for dihedral angle restraints). The distance restraints are checked for duplicates and are clustered into the following classes: intra-residual, sequential, medium-range (between 2 and 4 residues apart), long-range, or ambiguous.

#### QUEENY

A simplified and faster Python-based QUEEN (Nabuurs et al. 2003) implementation (called QUEENY) for residue-restraint information calculation was integrated within CING. It finishes well within a computer core minute for a 56 amino acid protein on regular hardware resources. The total per-residue restraint information is calculated and archived in the CING data structure.

### CING tools for structure analyses

The CING package implements several tools that aid the analysis of the structural results.

#### 2D dihedral angle combinations plots

Visualization of statistical preferences of dihedral angles provides information that can aid the assessment of the conformations in the structure ensemble. In CING, a large set of high-resolution X-ray structures were used to derive residue-specific statistical preferences for the dihedral angle combinations of Φ/Ψ (Ramachandran plot), χ^1^/χ^2^ [so-called Janin plot (Janin et al. 1978)] and the plot of the virtual dihedrals D1 and D2 (D1D2 plots) (see below). The reference dataset for the Ramachandran and Janin plots was based on the PDBSELECT database (v.2009-02-28) containing a set of 4,906 entries (5,135 chains and 88,540 residues) for which the R factor is <0.19 and the X-ray resolution better then 1.3 Å (Joosten et al. 2011). Background colouring in the Ramachandran and Janin plots was based on the DSSP classification (Hooft et al. 1996a) into helix (blue), sheet (yellow) and other (green). The colour changes linearly from white to e.g. yellow for densities from 2 to 20 %.

The virtual dihedral D1 of residue *i* is defined as the angle between the (non-)bonded atoms of residues *i* − 1 and *i*: C_*i*−1_^β^–C_*i*−1_^α^–C_*i*_^α^–C_*i*_^β^, whereas D2 is defined by the (non-)bonded atoms of residues *i* + 1 and *i*: C_*i*_^β^–C_*i*_^α^–C_*i*+1_^α^–C_*i*+1_^β^, so that that the D2 dihedral of residue *i* is identical to the D1 dihedral for residue *i* + 1. For glycine residues, which lack a β carbon, the H^α3^ is used instead of the C^β^. The D1 dihedral measures in one parameter the overall direction of the backbone over a two-residue segment. Four hundred residue-specific D1 distributions (20*20) were generated using a total of 1,044,392 amino acids that were selected from PDBSELECT entries with an R factor ≤0.21 and a resolution ≤2.0 Å. If the glycine H^α3^ atom was missing from the crystal structure, it was added on the basis of covalent geometry using the program YASARA. The D1D2 plot was then constructed as a 2D combination plot, assuming no correlation between the individual two-residue distributions, with D1 on the *x*-axis and D2 on the *y*-axis and gives an impression of the backbone direction over a three-residue segment. The D1D2 preferences for all 8,000 (20*20*20) D1D2 plots were binned and analysed by secondary structure as previously described for the Ramachandran plot (Hooft et al. 1997). The resulting plots (cf. Supplementary Fig. 2 for examples) show significant variations in the allowed regions and distributions of the different secondary structural elements, colour coded as in the Ramachandran and Janin plots.

**Fig. 2:**
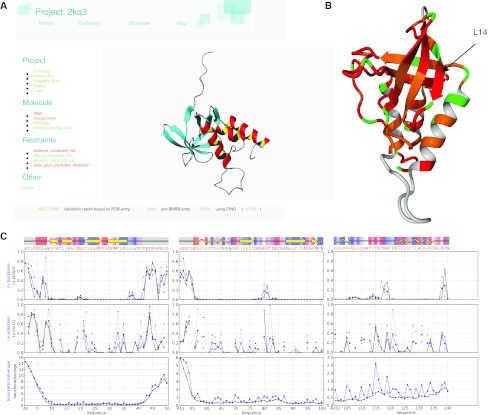
Overview of the CING analysis for PDB entry 2kq3 (Wang et al. 2010). **a** Project page of entry 2kq3. **b** Ribbon representation of the first conformer of the 2kq3 ensemble, colour-coded according to residue ROG score. Residues not included by the range selection are coloured grey. **c** Per-residue circular variance and positional RMSD values as function of residue number. DSSP-derived secondary structure analysis is shown on *top*. *Coloured bars* indicate relative solvent accessibility of each residue, as calculated by the WHAT_CHECK’s INOCHK routine, where *red* indicates “more exposed than usual” and *blue* means “more buried than usual”. Residues not included by the range selection are coloured *grey*

#### Outlier analysis

NMR typically generates ensembles of 20+ structures, so ‘outliers’ or spurious data present a serious problem for any measure that relies on averaging over the data points. CING uses an automated method based on Peirce’s criterion (Ross 2003) for outlier analysis and annotates the distributions of parameters, such as the dihedral angle distributions of individual residues. The models that fall outside the derived distribution are indicated in the text of the report and are colour coded in the corresponding plot. The original Peirce criterion code was extended to allow for analysis of larger data sets and limiting the number of outliers to be less than half of the complete set.

#### Range definition and superposition

For many validation criteria it is important to include only those residues that are in well-defined regions and many range definitions have been proposed in software tools such as: ARIA (Nilges et al. 1987), AQUA (Doreleijers et al. 1998), FindCore (Snyder and Montelione 2005), PDBstat (Bhattacharya et al. 2007), and UNIO (Guerry and Herrmann 2012). In CING, the range is assumed to be the full sequence when only one model is available or when the molecule contains no amino acids. The range includes those residues that have a Φ/Ψ dihedral angle circular variance (cv) of no more than 0.2. Consecutively, where the above selection caused a gap of four residues or less, those residues are reintroduced into the range. Short fragments of four residues or less are then omitted from the range. When this procedure results in an empty range, then the range is reset to all residues. Upon user request, CING can use range definitions that are based on chemical-shift derived order parameters (Berjanskii and Wishart 2008).

Using either user-specified or automatically determined ranges, the different conformers can be superimposed using backbone only or all heavy-atom selections. The RMSD to the average structure is then determined and the conformer closest to this average is reported.

#### Disulfide bonds

CING performs an analysis for the presence of potential disulfide bonds based on the coordinate data using the algorithm described by (Dombkowski and Crippen 2000; Pellequer and Chen 2006).

#### Salt bridges

Salt bridges cannot directly be inferred from experimental NMR data and therefore are established indirectly from the analysis of the coordinate data. Due to the sparse density of protons surrounding a typical salt bridge, the exact geometry often is not observed in every model of the ensemble. CING employs a classification proposed by (Kumar and Nussinov 2002) and reports on all combinations of potential salt bridge forming residue pairs (any Arg/Lys with any Glu/Asp).

### CING integration with external programs

By means of plugins (*vide supra*), CING integrates the analysis results of the external programs (cf. Table 1), with the most important ones detailed below.

#### PROCHECK_NMR

Even though the software package PROCHECK_NMR (Laskowski et al. 1996), like PROCHECK (PC), is no longer actively maintained, it has for a long time been the de facto validation standard for NMR spectroscopists. Even today, most papers quote the percentages of residues in the various regions of the PC Ramachandran plot. CING reports these numbers in its Summary page. The PC residue-specific G-factor is used in the CING ROG residues scores (cf. Table 2).

#### WHAT_CHECK

The software package WHAT IF contains an extensive subsection dedicated for structure validation which is available free of charge for academia under the name WHAT_CHECK (WC). A good introduction to the different WC checks is available at: http://swift.cmbi.ru.nl/gv/whatcheck. We have adapted the WC per-model and per-residue analyses results to ensemble properties suitable for validating an NMR ensemble.

CING reports on the overall WC scores on its Summary page and the following residue-specific WC properties on the Molecule page: (a) packing quality, Ramachandran, backbone normality, χ^1^, χ^2^ rotamers (Janin), χ^1^ rotamer (WC codes: QUA, RAM, BBC, C12, ROT, respectively), (b) bond lengths, bond angles, 2nd generation packing quality, protein side chain planarities, connections to aromatic rings, side chain planarity with hydrogens attached (BND, ANG, NQA, PLN, PL2, PL3, respectively), and (c) bumps, relative accessibility, accessibility, flip HIS GLN ASN hydrogen-bonds, torsion angle (BMP, ACCLST, INO, FLP, CHI, respectively). Only the Ramachandran and Janin WC Z-scores are used for ROG scoring in CING (cf. Table 2).

#### DSSP

The secondary structure elements were identified in each model of the ensemble by DSSP (Kabsch and Sander 1983) currently maintained in our laboratory (Joosten et al. 2011). The DSSP codes are collapsed in CING to three states of helix (DSSP: 3/H), sheet (DSSP: B/E), and other (all other DSSP codes). The state having the largest fraction of the three states in the ensemble is used as the overall consensus state.

#### Wattos

The inter-residue NOE distance restraint completeness up to 4 Å is analysed using Wattos (Doreleijers et al. 2005). The counts of observable atoms per residue and the expected, observed, and matched distance restraints are plotted for reference. The overall NOE completeness statistics are presented in the CING summary.

## Results

The two key concepts of the CING approach are the notions of a ‘Project’ and a residue-based analysis philosophy encompassing both experimental data and structure results. The project constitutes the complete collection of peaks, assignments, a molecule with chains, residues and atoms, all restraints, the coordinates of the structure ensemble and all the results of the validation routines and programs. These elements are linked according to the logical relationships that exist between them. For example, the project links to lists of restraints; each of these restraints links to its validation results as well as to the specific atoms involved (e.g. for a NOE), which in turn links to a residue, which links to a molecule. The reverse links are also modelled: project to molecule to chain to residue to atom etc. It is the presence of such linkages that makes the implementation of specific tasks or tests much simpler when compared to implementing them from scratch. The concept of a Project also allows for an easy connection between information originating from different programs.

The CING report consists of a collection of HTML/Web 2.0 pages that reflect the hierarchy of the project and the links between the entities. Thus, the pages provide for easy navigation between structure and data elements and all entities within the CING project are coloured according to their three-way ROG-assessment.

NMR structures often contain unstructured regions. A crucial aspect of any validation therefore concerns the decision which residues of the biomolecule to include in the assessments. To test the range-selection criterion, we selected from 9,300 NMR NRG-CING entries (Doreleijers et al. 2011) those entries of at least 30 amino acids and 10 structure models. This yielded 6,460 entries encompassing 7,735 chains, 10,088 segments and 624,958 residues. Using the CING circular variance-based range selection analysis (described above), overall only 13 % of the residues are excluded. The mean ordered segment was 61.9 residues long and there were on average 1.3 segments per polypeptide chain. The average number of segments per chain is considerably reduced from the 3.2 that we previously found using a simple window averaging scheme (Doreleijers et al. 1998). This new procedure produces similar results as the consensus procedure of PSVS (Bhattacharya et al. 2007) used in the 2010 CASD-NMR assessment (Rosato et al. 2012). The CING-derived ranges of the CASD-NMR targets include a total of 921 residues of which 75 residues were excluded by PSVS (data not shown). Conversely, there are only 5 residues that are not in the CING derived ranges. For example, the CING range for PDB entry 2kpm (unpublished) is [10–98]. It includes 29 amino acids at the termini that were excluded in the CASD-NMR range [23–82]. The excluded residues 83–98 display low backbone flexibility (c.v. of 0.009) and 44.8 distance restraints per residue on average, suggesting that they could and perhaps should have been validated.

### Example CING report

The recently submitted PDB entry 2kq3 comprises the NMR-derived structure ensemble (20 models of 140 amino acids) of the monomeric and very well-studied staphylococcal nuclease protein. The NMR ensemble was obtained using 2,089 distance restraints, 64 hydrogen-bond restraints and 147 dihedral restraints (Wang et al. 2010). The ensemble and its experimental dataset are typical for NMR-derived proteins of this size; it was chosen at random from a set of entries with similar properties.

Figure 2 shows parts of the CING report for 2kq3. The full CING analysis of 2kq3, including many more figures than the ones displayed in this manuscript, can be obtained from http://nmr.cmbi.ru.nl/NRG-CING/data/kq/2kq3/2kq3.cing. The Project page (cf. Fig. 2a) is the starting point for the report and shows a first impression of the monomeric protein with the beta sheet and alpha helices as well as the colour-coded entities to the left.

The CING Summary page reports on the overall CING, WHAT_CHECK and PROCHECK scores as well as the structural variation analysis (data not shown). Of the 140 residues of the polypeptide, the CING analysis identified 122 residues to be structured and excluded the four disordered N-terminal residues and an unstructured loop for residues 42–55 (top panel of Fig. 2c). The structured residues have an RMSD to the mean of 0.96 ± 0.21 Å for backbone atoms only. The values reported by Wang et al. on the basis of secondary structure are considerable less, i.e. 0.32 ± 0.07 Å (Wang et al., Supplementary Table 1), suggesting a much tighter bundle. The CING Summary page also lists overall WHAT_CHECK and PROCHECK results; Wang et al. also reported the latter (Supplementary Table 1), and comparison between the CING (78/19/3/0%) and original (72/23/3/2%) analysis show that the overall PROCHECK scores are similar. The average WHAT_CHECK Ramachandran and rotamer normality scores (−5.1 and −7.1) reported by CING are however strongly indicative of poor conformations. The Summary page reports that 55 out of the 122 structured residues (45 %) have been flagged ‘red’ for the CING ROG score and 46 residues (38 %) orange. When the ROG scores are mapped onto the structure (Fig. 2b), they indicate that the problems and the warnings encompass nearly the whole protein. The RECOORD protocol for recalculation and subsequent refinement in water results in a much improved ensemble for many PDB entries (Nederveen et al. 2005). After applying the protocol to this entry, the number of residues marked red dropped from 55 to 28 % and the WHAT_CHECK Ramachandran and rotamer normality scores improved from −5.1 and −7.1 to −2.6 and −4.2, respectively. The core backbone atom pairwise RMSDs between the original and recalculated ensembles is 1.5 ± 0.2 which is higher than the variance within the recalculated ensemble (1.2 ± 0.3), indicating a significant structural adjustments. Wang et al. were not available for comments on these findings.

**Fig. 3:**
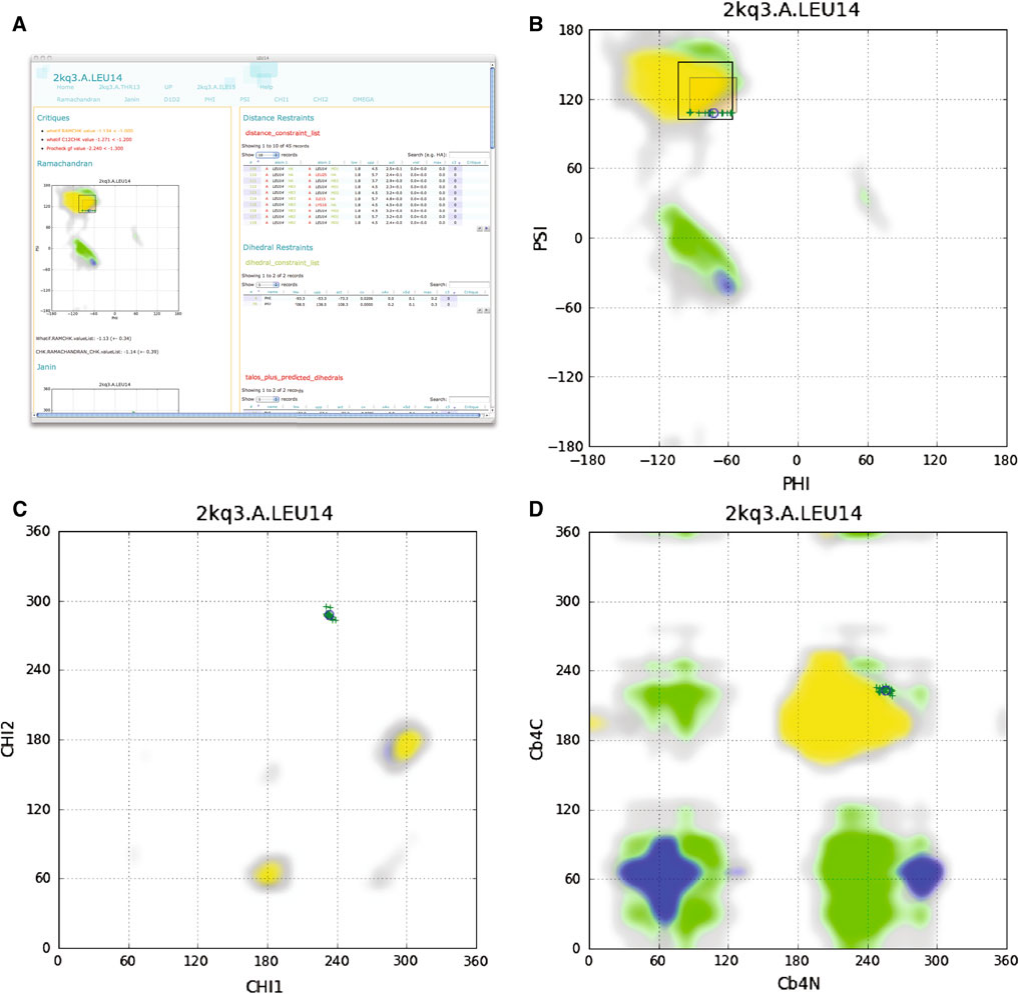
Residue analysis of 2kq3 residue Leu14. **a** The CING Residue page for Leu14. Structure analysis is displayed on the *left* and shown in detail in panels (**b**)–(**d**); experimental data involving Leu14 shown on the *right*. **b** Ramachandran plot of Leu14. Residue specific background colouring derived from the WHAT_CHECK protein reference database for helical (*blue*); β-sheet (*yellow*) and coil (*green*) regions as determined by DSSP. *Grey areas* define low-density transition regions. Experimental ϕ/ψ restraints are indicated by the *transparent orange box*. Experimental ϕ/ψ values of the individual members of the ensemble are indicated by *green plus signs*; its average value by an *open circle*. *Open square box* denotes an automatically Talos+ derived ϕ/ψ region on the basis of the experimental chemical shifts. **c** Janin plot of Leu14; colouring as in (**b**). D) D1D2 plot (see text) of Leu14; colouring as in (**b**)

The Residue page for residue Leu14 of entry 2kq3 is shown in Fig. 3a. On the left-hand side, CING first lists four critiques by WHAT_CHECK, CING and/or PROCHECK-NMR (Fig. 3a). The individual conformers of the ensemble are displayed below the critiques by means of the Ramachandran, Janin, and D1D2 plot (Fig. 3b–d, respectively). The underlying colouring of these plots is derived from the residue-specific analysis of the WHAT_CHECK database of high-resolution X-ray crystal structures (see “Methods”) and allows for a visual assessment of the likelihood of the observed conformations. The right-hand side of the Residue page tabulates the experimental restraints that involve atoms of Leu14. These tabular entries are expandable, searchable, can be sorted on any column and are hyperlinked to the corresponding Atom and Residue pages.

Oddly, the Ramachandran plot of Leu14 (Fig. 3b) shows all conformers of the ensemble to cluster near a single ψ value of ~109°, which appears to be dictated by the dihedral restraint for this angle (indicated by the transparent orange box). The experimental Φ,Ψ values of the conformers are also close to the edge of the commonly observed conformations for Leucine, resulting in relatively poor WHAT_CHECK score and PROCHECK G-factor. The Janin plot (Fig. 3c) shows the side chain of Leu14 to be tightly restricted in a staggered χ_1_ conformation, which also appears unlikely from the database reference distribution. Finally, the D1D2 plot shows the conformation of Leu14 with respect to its previous and next residue to be in the extended conformation, albeit at the edge of what is commonly observed. Hence, the ‘red’ ROG score for Leu14 originates from this set of poor conformations.

Leucine 14 is part of a stretch of poorly modelled residues in this first β-strand of staphylococcal Nuclease, and only Ala17 in this strand has a green ROG score. For example, the backbone dihedral angles of Asp19 cluster in a very unfavourable region of the Ramachandran plot (Supplementary Figure S1). This conformation also conflicts with the Φ,Ψ dihedral restraint region derived by the CING Talos+ analysis (shown as an open box in Supplementary Figure S1), on the basis of the experimental chemical shift data. Potentially, the poor conformations of Leu14, and the directly following troublesome β-bulge residues Leu15 and Lys16, are the result of a set of smaller but propagating and reinforcing errors. The analysis of the conformation of residues and identification of potential problems can conveniently be done using the ‘Dihedral plots per residue’ page, which displays the relevant plot of all residues sequentially, in one scrollable interface (Supplementary Figure S2).

#### Restraint validation

The agreement between the restraints and the resulting structure ensemble is a commonly calculated parameter for NMR structures. The numbers of restraints are tabulated and their RMSD and other violation statistics are typically reported. CING also reports the results of such a full restraint analysis.

**Fig. 4:**
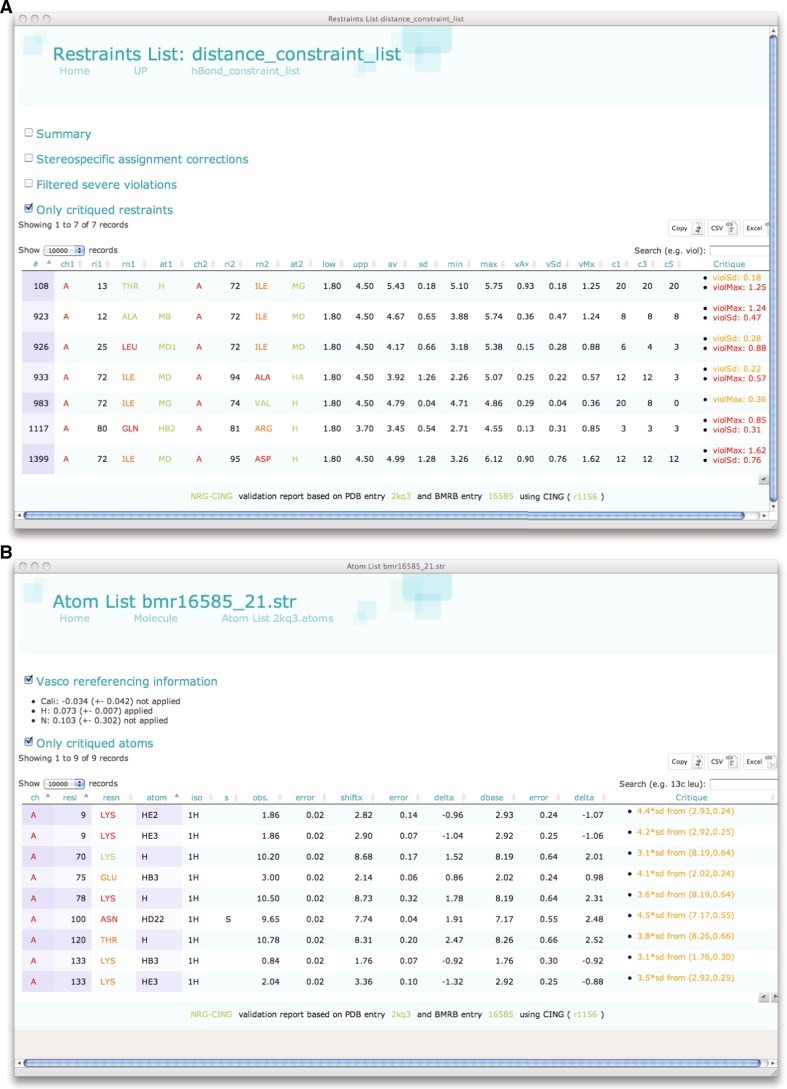
CING analysis of experimental data. Expandable, searchable, tabular displays are used, that can be sorted on any column. Table entries are directly hyperlinked to the corresponding Residue and Atom pages. **a** ‘Distance restraints’ page. *Check boxes* allow for additional information to be displayed. Only critiqued restraints are shown. **b** Atoms page. Only critiqued atoms are shown

There are several issues with entry 2kq3, which unfortunately are quite common in current PDB entries. In the supplementary material Table S1 of the paper describing the 2kq3 structure (Wang et al. 2010) the authors report zero distance restraint violations above 0.3 Å. The CING analysis, however, shows five troublesome restraints (Fig. 4a), in addition to two severe violations that were already filtered out during the restraint mediation. Moreover, out of the total of 2,089 non hydrogen-bond distance restraints, the CING analysis identifies 298 duplicates. Despite a reasonably high number of distance restraints per residue, the CING Wattos analysis reports a low overall NOE completeness of 32 % compared to the NRG database average of 57 %. This observation suggests that the data content used in this structure determination was of below average quality.

#### Chemical shift validation

The Atom page reports on CING’s comparisons of the experimental chemical shifts, after VASCO re-referencing (Rieping and Vranken 2010), with both the BMRB database values and the back-calculated values from the coordinates using the SHIFTX software (Zhang et al. 2003). A report for PDB entry 2kq3 and corresponding BMRB entry 16585 is shown in Fig. 4b. A small 0.07 ppm offset was determined by VASCO for the ^1^H nuclei and applied automatically. ^13^C and ^15^N corrections were minor, i.e. −0.03 and 0.1, respectively and not applied because the corrections were less than three times the uncertainty. No assignment consistency issues were detected by CING.

The comparison of the experimental values with the database values is the basis for detecting outliers for ROG scoring. For 2kq3 CING identifies nine instances in which the experimental values deviate by more than 3 standard deviations from their database averages (cf. Fig. 4b). As an example, one of the most extreme cases involves the H^δ22^ side chain atom of Asn100, which is listed to resonate at 9.65, 2.5 ppm (4.5 standard deviations) higher then its average value. The experimental value is also 1.9 ppm higher than its SHIFTX predicted value. Whereas the H^δ12^ atom is involved in two long-range restraints to the amide protons of Ile92 and Leu37, no restraints involving the H^δ22^ atom are included in the restraint set. Intra-residue or sequential restraints involving these atoms are also not present in the dataset. The χ1/χ2 side chain angle value-pairs of Asn100 cluster in two, non-ideal regions of the Janin plot and the H^δ21^ and H^δ22^ are packed in a hydrophobic environment. Many NOEs, also involving the H^δ22^ atom, should have been observable in such a conformation and thus should have resulted in restraints. In all, the extreme H^δ22^ experimental chemical shift value and the combined pattern of distance restraints and structural conformation, strongly suggests an erroneous assignment for one or more of the atoms of Asn100.

### Salt bridges

As an example of the salt bridge detecting functionality of CING, a water-refined ensemble of structures of the second domain (CBD2) of NCX under Ca^2+^-free conditions (PDB entry 2kls) was examined (Hilge et al. 2009). In the apo structure, three basic residues in CBD2, Arg547, Lys583, and Lys585, are well positioned to form salt bridges with Asp552, Asp578, Glu580, and Glu582. For example, the CING analysis showed Lys585 to engage in an interaction with Asp552, classified as salt bridge in 18 out of 20 models. In addition, electrostatic interactions of Lys585 to Asp578 and Glu582 were identified as salt bridges (7/8 out of 20 models, respectively) or ionic interactions (10/11 out of 20 models). These now unambiguously identified electrostatic interactions were previously shown to be crucial, as they partly stabilize some of the negative charges resulting from the release of Ca^2+^ and therefore prevent unfolding (Hilge et al. 2006).

### Comparison between CING and PROCHECK_NMR

**Fig. 5:**
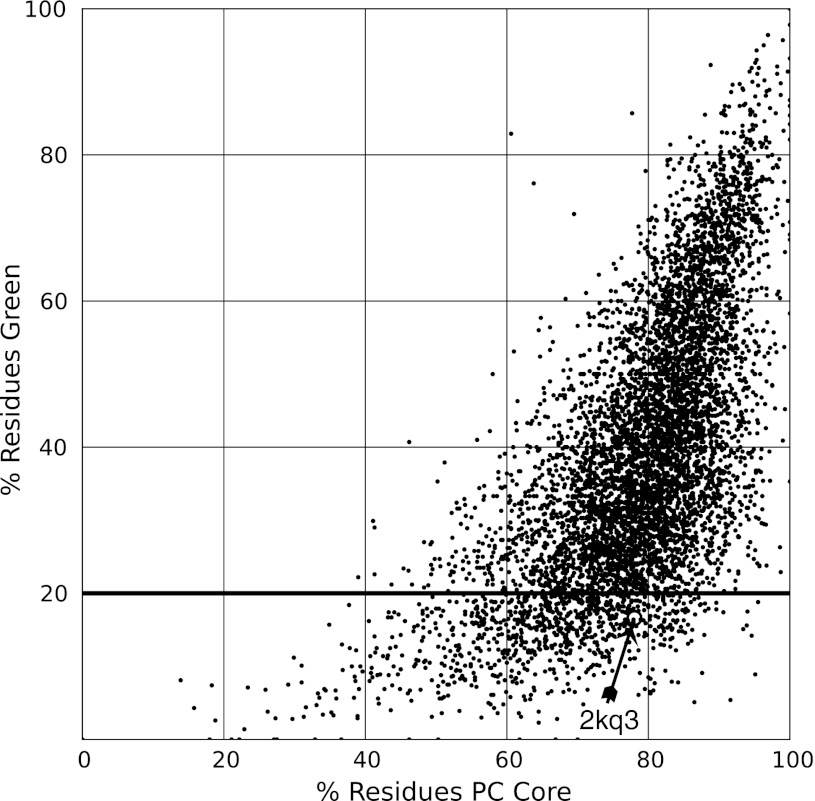
Comparison of the fraction of well-defined residues with a CING ROG score green (good) versus the fraction of residues that fall in the most favoured region of the Ramachandran plot according to PROCHECK-NMR. The *horizontal line* at 20 % indicates the CING cut off for the minimum percentage of *green residues*, beyond which the molecule as a whole is flagged red regardless of the allocation of orange and *red residues* (see Table 2). The *plot* shows a significant number of entries on the *bottom right* for which a high percentage of ‘PC core’ residues are not ‘*green*’ in CING. Data is shown for the 6,383 NMR PDB entries selected from NRG-CING to have at least ten models and 30 or more amino acid residues. Indicated with an *open circle* (at 77.9 %) is PDB entry 2kq3 that is discussed as an example in the text

For many years, PROCHECK_NMR has served as the de facto validation standard, although it has been clear for long that updates to its reference values were needed. The most quoted validation criteria today are the PROCHECK_NMR Ramachandran plot percentages of residues in the regions denoted core, allowed, additionally allowed, and disallowed. To test the correlation between the PROCHECK_NMR and CING ROG scores, the percentage of residues in the core region is plotted versus the CING percentage of green residues (Fig. 5).

Analysis of the data indicates that a significant number of entries combine high PROCHECK_NMR scores (>75 %) with low ROG green scores, which flags these as highly suspect. The entry 2kq3 (critiqued above) also combines a 78 % PROCHECK_NMR score with a very low 17 % ROG green score. Conversely, there are almost no entries in the top left corner of the plot that would be entries found to be problematic by PROCHECK_NMR but not by CING (top left corner of the plot), which is to be expected as the CING ROG score incorporates the residue-specific PROCHECK_NMR G-factor.

## Discussion

A residue is a natural concept for NMR-based structure determination. NMR assignment strategies are almost exclusively residue-based. NMR related parameters are residue type dependent and the local nature of the NMR-derived restraints also correlates well with a residue-based approach. Structural properties can also be conveniently summarized at the residue level. We previously showed that structurally bad regions are masked when using overall validation parameters (Nabuurs et al. 2006), which can be circumvented by a residue-based approach.

The 2kq3 structure was chosen as a typical representative of an NMR structure in terms of molecular size and experimental restraints. Distance and dihedral restraints still constitute the input data for the majority of the NMR ensembles, including entry 2kq3, while RDC restraints have only been deposited for only 499 of the >9,000 NMR-derived structures (Doreleijers et al. 2011). The 2kq3 entry has a high percentage of residues that CING flags as ‘red’ or ‘orange’ (Fig. 2b), as a result of many uncommon backbone or side chain dihedral angles (Fig. 3; Supplementary Figures S1, S2). The ROG scores combines the analysis of several tools (cf. Table 2) and is effective in flagging problematic regions. Comparison of the dihedral restraints deposited by the authors and the Φ/Ψ dihedral restraint region derived by the CING Talos+ analysis, shows the former to be significantly more restricted and often to result in suspect Φ/Ψ angle distributions. In addition, the overall information content appears low on the basis of the Wattos NOE completeness criterion and we identified several suspect assignments (Fig. 4). These problems warrant a careful inspection of the original data and derived restraints and we established that a refinement of the structure ensemble using an extended force field that included electrostatics and water yields better results.

The CING analysis is sensitive to local problems and ill-refined structures rapidly result in large numbers of red or orange flagged residues. However, in absence of any gross restraint errors and using a proper water-refinement protocol (Spronk et al. 2002), the local errors can be remedied readily and the green scores improved. For example, the pre-water refined NCX3-CBD2-B ensemble of structures obtained from CYANA calculations yielded ROG scores of 53/30/17%, but improved to 23/31/46% after refinement in explicit solvent using the YASARA YAMER force field (Breukels et al. 2012). Similar improvements in structure quality were obtained in the DRESS (Nabuurs et al. 2004) and RECOORD (Nederveen et al. 2005) databases of recalculated and refined NMR structures.

In our experience, based on spot checking dozens of entries in NRG-CING, a properly refined ensemble of structures that has low green ROG (<~20 %) and high red ROG (>~50 %) scores can be labelled as highly troublesome. Entry 2kq3 showed numerous issues, in spite of its relatively high PROCHECK_NMR scores, which illustrates the latter to be a less reliable indicator of structure quality. Multiple parameters, as implemented in the CING ROG scores, appear more sensitive to identification of potentially problematic structures. We have not manually examined all entries that combine high PROCHECK_NMR scores with low ROG green scores (cf. Fig. 5), but for those entries that were examined clear problems could typically be identified.

It is important to keep in mind that a substantial part of the CING analysis and resulting ROG scores is based upon comparisons with database-derived properties. Particular features of a structure not present in the reference databases will therefore be flagged as red or orange, in spite of these potentially being correct. However, given the now extensive nature of the structure database, such occurrences are very rare and should be treated with extreme caution. Examples of these are the inclusion of unusual amino acids or chemical modification of residues.

In the case of the now retracted entry 1tgq, which prompted the development of the CING suite, the analysis clearly shows major problems (ROG scores 54/30/16%) (Nabuurs et al. 2006). In addition, back-calculated chemical shifts (data not shown) readily identify the troublesome kinked α-helical region and the errors for β-strand 3. In contrast, the correct 1y4o structure (Song et al. 2005) displays normal ROG scores (16/27/57%).

One category of ill-folded structures that are not recognised by CING are those derived using the chemical-shift based ROSETTA protocol (Shen et al. 2008) or its variants. As the CS-ROSETTA protocol samples from a structure database and uses chemical shift matching for fragment selection, it optimises the two main criteria that CING uses for identification of problems. It is therefore not surprising that analysis of the automated structure calculation efforts of CASD-NMR (Rosato et al. 2009) showed the CING routines unable to identify the incorrectly folded CS-ROSETTA derived structures (Rosato et al. 2012).

In general, a proper validation assessment should be based on information that is not used to calculate the structure ensemble. Such cross-validation procedures are now feasible for most high-resolution NMR derived structures because the information content of the restraints used for typical structure calculations has greatly increased over the past decade, and part of them could be left out to validate the results.

## Conclusions

This paper describes an integrated residue-based approach for NMR structure validation, yielding validation reports for authors, referees, and end-users. The intuitive red-orange-green set of residue-based critiques directs the attention to specific parts of the structure in need of manual verification. The iCing server and the CCPN analysis program allow for straightforward upload and initial visualization of the validation results, enabling individual users to test their structure ensemble prior to submission to the PDB and its reporting in a manuscript. The CING validation suite was developed for, but is not limited to, NMR-derived protein structures. Oligonucleotide and X-ray structures can also easily be examined, albeit that no experimental X-ray data can be validated for the latter. The CING suite will continue to evolve, also in response to recommendations put forward by the wwPDB NMR validation taskforce.

Finally, the *Journal of Biomolecular NMR* like many journals, requires authors of new structure papers to deposit the coordinate and experimental data in accordance to the IUPAC guidelines (Markley et al. 1998). However, referees are not usually provided with any external validation report on those coordinates. It would be of great value to authors and referees to have the CING reports available as part of their submissions.

## Electronic supplementary material

Below is the link to the electronic supplementary material.

Supplementary material 1 (PDF 907 kb)
