# Supplementary material for: CING: an integrated residue-based structure validation program suite
Source: J Biomol NMR. 2012 Sep 18;54(3):267–83. doi: 10.1007/s10858-012-9669-7 (PMC3483101; doi:10.1007/s10858-012-9669-7)
Supplement: Supplementary file 1 — Supplementary material 1 (PDF 907 kb) [file 10858_2012_9669_MOESM1_ESM.pdf]

CING; an integrated residue-based structure validation program suite

Supplementary Materials

Jurgen F. Doreleijers[1], Alan W. Sousa da Silva[2], Elmar Krieger[3], Sander B. Nabuurs[1], Christian A.E.M. Spronk[4], Tim J. Stevens[5], Wim F. Vranken[6,7], Gert Vriend[1] and Geerten W. Vuister[8,*]

1 CMBI, Radboud University Medical Centre, Geert Grooteplein 26-28, 6525 GA, Nijmegen, The Netherlands.

2 UniProt, European Bioinformatics Institute, Hinxton, Cambridge, CB10 1SD, United Kingdom.

3 YASARA Biosciences GmbH, Wagramer Strasse 25/3/45, 1220 Vienna, Austria.

4 Spronk NMR Consultancy UAB, Palangos gatvė 4 LT-01402, Vilnius, Lithuania.

5 Department of Biochemistry, University of Cambridge, 80 Tennis Court Road, Cambridge, CB2 1GA, United Kingdom.

6 Department of Structural Biology, VIB, Building E, 4th Floor, Pleinlaan 2, 1050 Brussels, Belgium.

7 Structural Biology Brussels, Vrije Universiteit Brussel, Building E, 4th Floor, Pleinlaan 2, 1050 Brussels, Belgium.

8 Department of Biochemistry, Henry Wellcome Building, University of Leicester, Lancaster Road, Leicester LE1 9HN, United Kingdom.

* Corresponding author

Postal: Department of Biochemistry, Henry Wellcome Building, University of Leicester, Lancaster Road, Leicester LE1 9HN, United Kingdom.

Telephone/ Fax: +44 116 229 7076/+44 116 229 7018

E-mail: gv29@le.ac.uk

Keywords: NMR, Structure Validation, PDB, Errors, Quality, Protein Structure

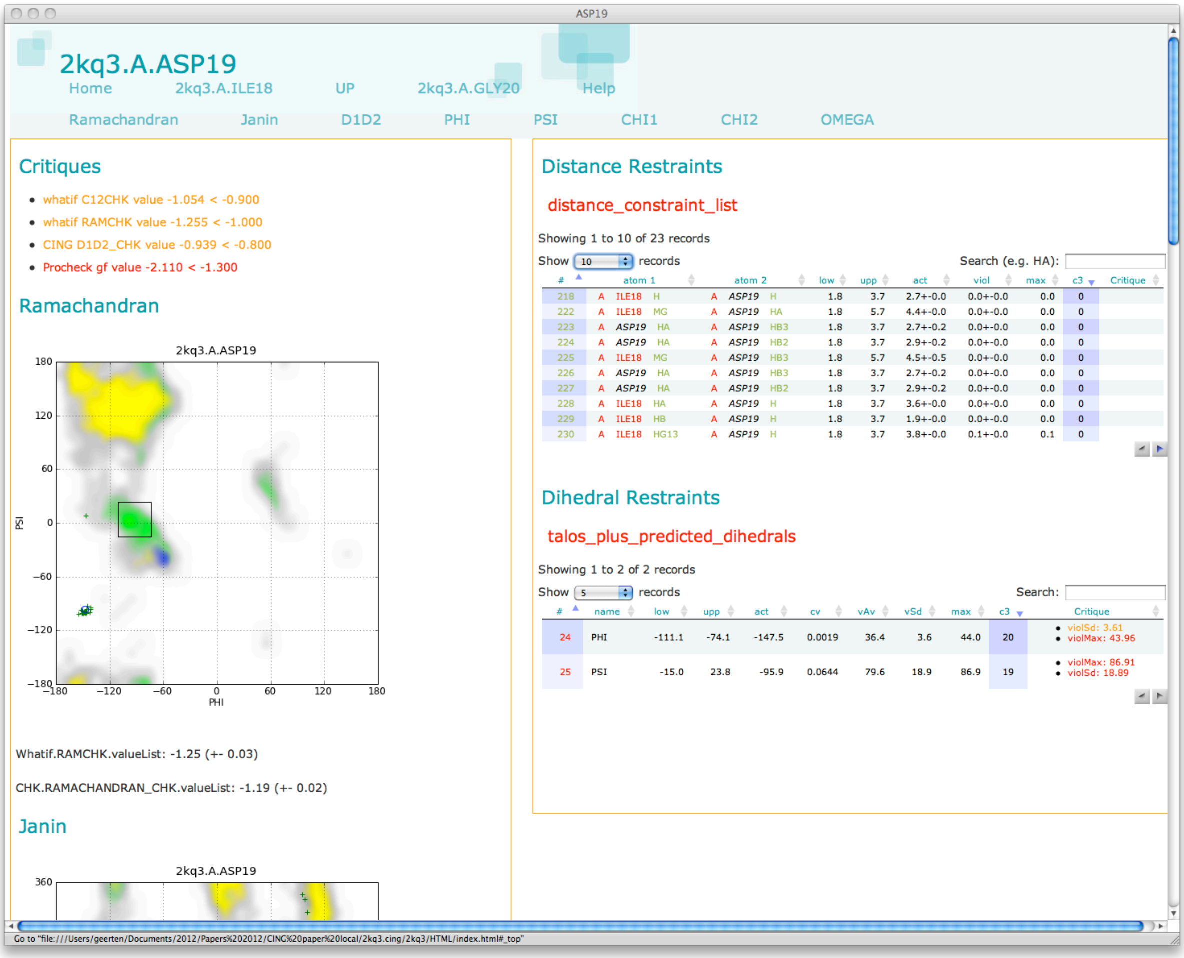

Supplementary Figure S1.
CING Residue page for PDB entry 2kq3 residue Asp19.

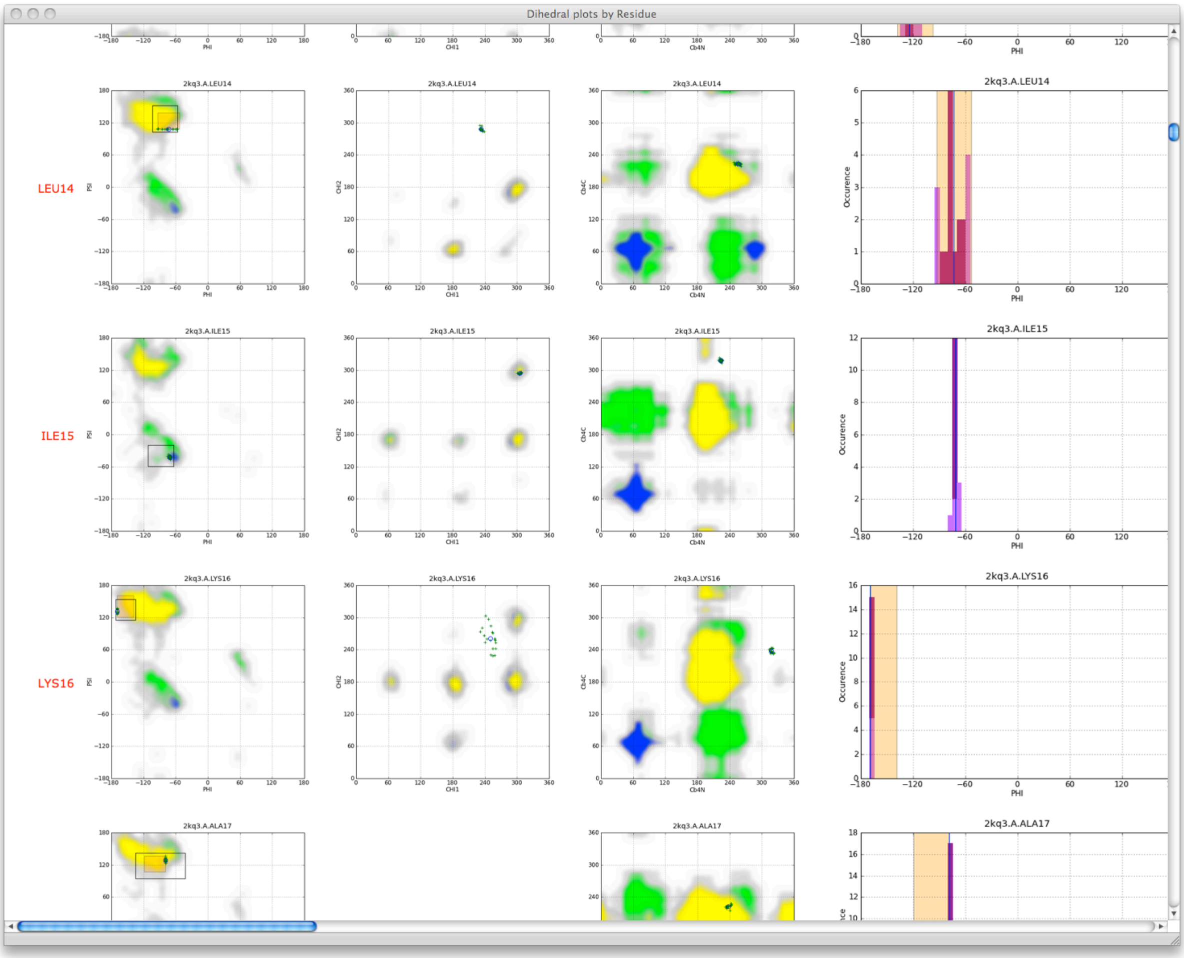

Supplementary Figure S2.
CING 'Dihedral plots by residue' page for PDB entry 2kq3, residues Leu14-Lys16.
